# Skin Microbiota in Obese Women at Risk for Surgical Site Infection After Cesarean Delivery

**DOI:** 10.1038/s41598-018-27134-5

**Published:** 2018-06-08

**Authors:** Kara M. Rood, Irina A. Buhimschi, Joseph A. Jurcisek, Taryn L. Summerfield, Guomao Zhao, William E. Ackerman, Weiwei Wang, R. Wolfgang Rumpf, Stephen F. Thung, Lauren O. Bakaletz, Catalin S. Buhimschi

**Affiliations:** 1 Department of Obstetrics and Gynecology, The Ohio State University College of Medicine, Columbus, Ohio 43210 USA; 2 Center for Perinatal Research, The Research Institute at Nationwide Children’s Hospital, Columbus, Ohio 43215 USA; 3 Department of Pediatrics, The Ohio State University College of Medicine, Columbus, Ohio 43210 USA; 4 Battelle Center for Mathematical Medicine, The Research Institute at Nationwide Children’s Hospital, Columbus, Ohio 43215 USA; 5 Center for Microbial Pathogenesis, The Research Institute at Nationwide Children’s Hospital, Columbus, Ohio 43215 USA

## Abstract

The obesity pandemic in the obstetrical population plus increased frequency of Cesarean delivery (CD) has increased vulnerability to surgical site infection (SSI). Here we characterized the microbiome at the site of skin incision before and after CD. Skin and relevant surgical sites were sampled before and after surgical antisepsis from obese (n = 31) and non-obese (n = 27) pregnant women. We quantified bacterial biomass by qPCR, microbial community composition by 16sRNA sequencing, assigned operational taxonomic units, and stained skin biopsies from incision for bacteria and biofilms. In obese women, incision site harbors significantly higher bacterial biomass of lower diversity. Phylum *Firmicutes* predominated over *Actinobacteria*, with phylotypes *Clostridales* and *Bacteroidales* over commensal *Staphylococcus* and *Propionbacterium* spp. Skin dysbiosis increased post-surgical prep and at end of surgery. Biofilms were identified post-prep in the majority (73%) of skin biopsies. At end of surgery, incision had significant gains in bacterial DNA and diversity, and obese women shared more genera with vagina and surgeon’s glove in CD. Our findings suggest microbiota at incision differs between obese and non-obese pregnant women, and changes throughout CD. An interaction between vaginal and cutaneous dysbiosis at the incision site may explain the a priori increased risk for SSI among obese pregnant women.

## Introduction

Obesity is a risk factor for Cesarean Delivery (CD)^1,2^ and is associated with a significantly higher risk of surgical site infection (SSI) including wound infections and post-partum endometritis^3–5^. Compared to women with a normal body mass index [(BMI) 18.5 to 25)], obese women (BMI > 30) are twice as likely to develop SSI post-CD^6^.

Medical, quality of care and financial incentives have generated pressure to reduce SSI rates for all surgical specialties^7^. Among universally effective strategies were the standardization of protocols for skin preparation and antisepsis and for use of preoperative antibiotics^8,9^. Despite their adoption in obstetrics, 6–10% of CDs continue to develop SSIs^7,10,11^.

Most species of bacteria that cause SSIs are part of the normal human microflora and form biofilms at sites where they exist as commensals^12^. Due to various factors that disturb local ecology biofilms that harbor bacteria they have been implicated as the cause of 65% of all microbial infections^13,14^. Microbiologic evidence the notion that biofilm bacterial communities demonstrate resistance to routine surgical preparation protocols^15,16^.Skin microbial community profiling and biofilm research have remained largely two separate scientific areas, but metagenomics has paved the road toward their convergence. Given the correlation between increasing body weight and higher risk for SSI, it was reasonable to propose that in obese pregnant women the anatomical characteristics of the panniculus fold create a unique microbiologic environment. To address this gap in knowledge we measured the bacterial biomass and microbial diversity and assessed for resence of biofilms in obese versus non-obese pregnant women. We further examined the effectiveness of a universally-recognized preoperative antisepsis protocol^17^ on commensal colonization at surgically relevant cutaneous sites prior to and after delivery of the newborn.

## Results

### Clinical Characteristics of the Study Population

For analysis purpose cases were separated into two groups: non-obese (BMI < 30, n = 27) and obese (BMI ≥ 3, n = 31, class 1: n = 11; class 2: n = 7; class 3: n = 13). Compared to normal BMI women, obese mothers had a higher pre-pregnancy BMI (*P* < 0.001), panniculus grade (*P* < 0.001) and a longer time period from showering before enrollment (*P* = 0.011). Within 14 days after Cesarean, 15% (9/58) of enrolled women developed an SSI (BMI < 30, n = 2 and BMI ≥ 30, n = 7, *P* = 0.111) Details about demographic and surgical characteristics of the groups are presented in Supplementary Table S1.

### Topographical and Temporal Changes in Bacterial Biomass

**Figure 1:**
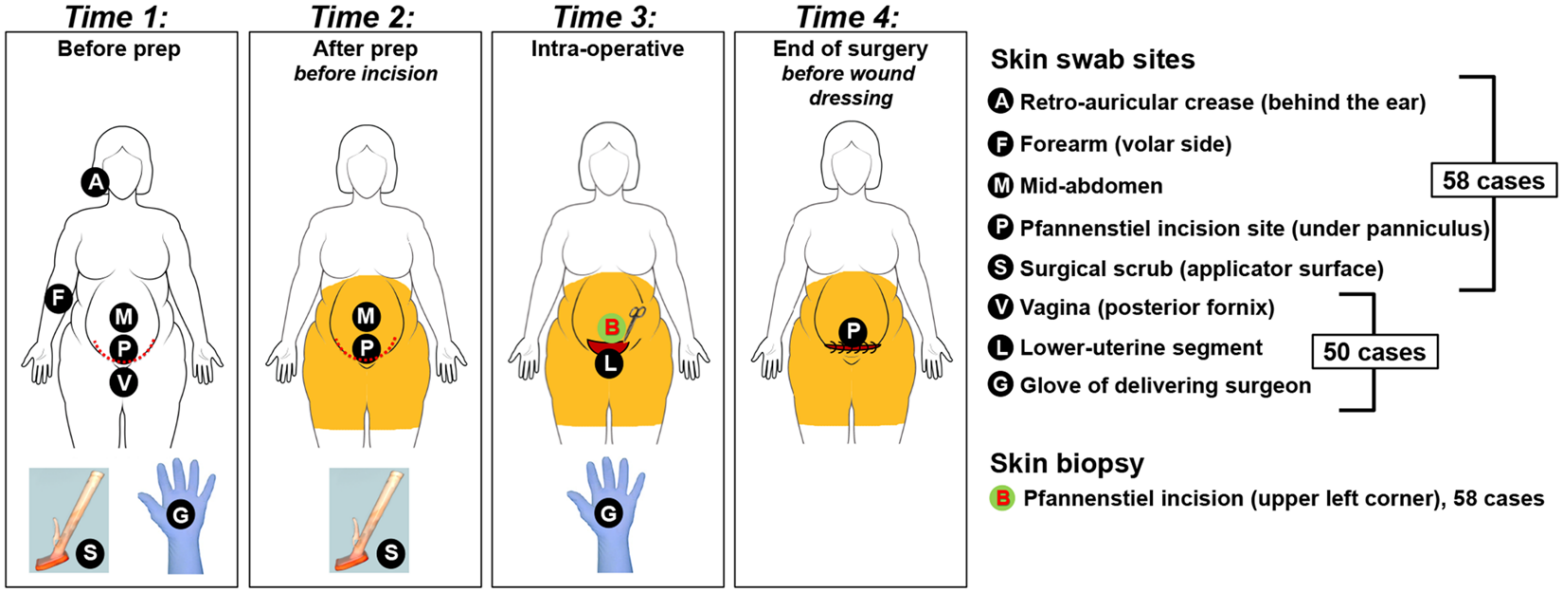
Schematic representation of the sampled sites relative to antisepsis and surgical times.

**Figure 2:**
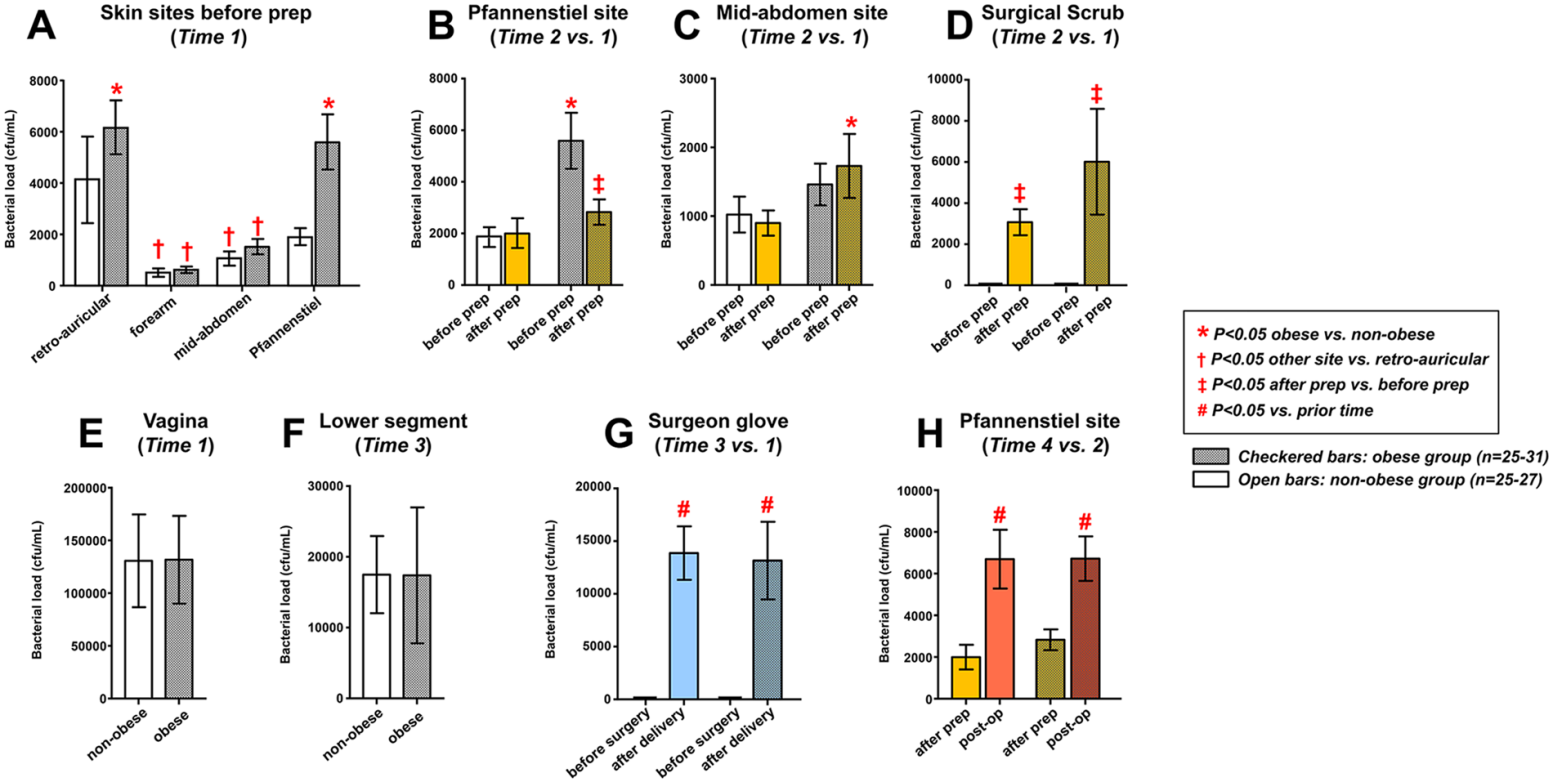
Topographical and temporal changes in bacterial load in obese and non-obese women undergoing scheduled Cesarean delivery. Bacterial load quantified from samples collected from skin sites before prep (**A**), Pfannenstiel site before and after prep (**B**), mid-abdomen (**C**) and surgical scrub (**D**) before and after prep, vagina (**E**) and forearm (**F**) before procedure, and surgeon glove before and after surgery (**G**) and after prep and post-op at Pfannenstiel site (**H**).

A schematic of the sampled sites relative to antisepsis and surgical times is provided in Fig. 1. Before surgical skin antisepsis, the highest bacterial loads for obese than for non-obese women were seen retro-auricular and in the Pfannenstiel area (Fig. 2A, *P* < 0.05). Compared to the mid-abdominal site, the average bacterial load at the Pfannenstiel site was 3.8-fold higher in obese group, but just 1.8-fold higher in the non-obese women.

Surgical antisepsis at Pfannenstiel site did not impact bacterial load in non-obese women (Fig. 2B, *P* = 0.917). Conversely, bacterial load at Pfannenstiel site was significantly reduced in obese women after prep (*P* = 0.006) to a point where there was no longer a difference between groups (*P* = 0.416). Obesity was characterized by an increase in bacterial load at the mid-abdominal site compared to non-obese women (Fig. 2C, *P* = 0.046). After skin prep, the bacterial load of the surgical scrub was significantly increased in both obese and non-obese women (Fig. 2D, *P* < 0.001). No differences were noted in vaginal bacterial load between obese and non-obese groups (Fig. 2E, *P* = 0.981). Vaginal bacterial load was notably higher (69-fold for non-obese and 24-fold for obese group vs. Pfannenstiel site) compared to the skin sites sampled at the same *Time 1*.

Sampling of the lower uterine segment after delivery of the baby showed this site is rich in bacteria in both obese and non-obese women with no differences between groups (Fig. 2F, *P* = 0.695). A similar pattern was observed on the surgical glove where the bacterial load was found to be significantly higher compared to pre-surgery (Fig. 2G, *P* < 0.001). The bacterial load of the glove and of the lower uterine segment at *Time 3* were similar for both obese (*P* = 0.582) and non-obese women (*P* = 0.698). At the end of surgery and following wound closure, the edges of the Pfannenstiel incision had a very high bacterial load compared to the skin area immediately after scrubbing (Fig. 2H, *P* < 0.001). The level of added bacteria was similar for obese and non-obese groups (*P* = 0.872). Bacterial load at the Pfannenstiel incision after the procedure was determined by bacterial load of the lower uterine segment (multivariate regressions, R^2^ = 0.530, *P* < 0.001) but not by BMI, thickness of subcutaneous tissue, race or bacterial loads of the vagina or Pfannenstiel area after prep (*P* > 0.100).

### Topographical and Temporal Changes in Microbial Communities at Phylum Level

Preprocessing of raw sequence data yielded a total of 1,998,254 high quality sequences which mapped to 452 genera. These converged taxonomically into 31 distinct phyla of which only one had status *Candidatus*.

**Figure 3:**
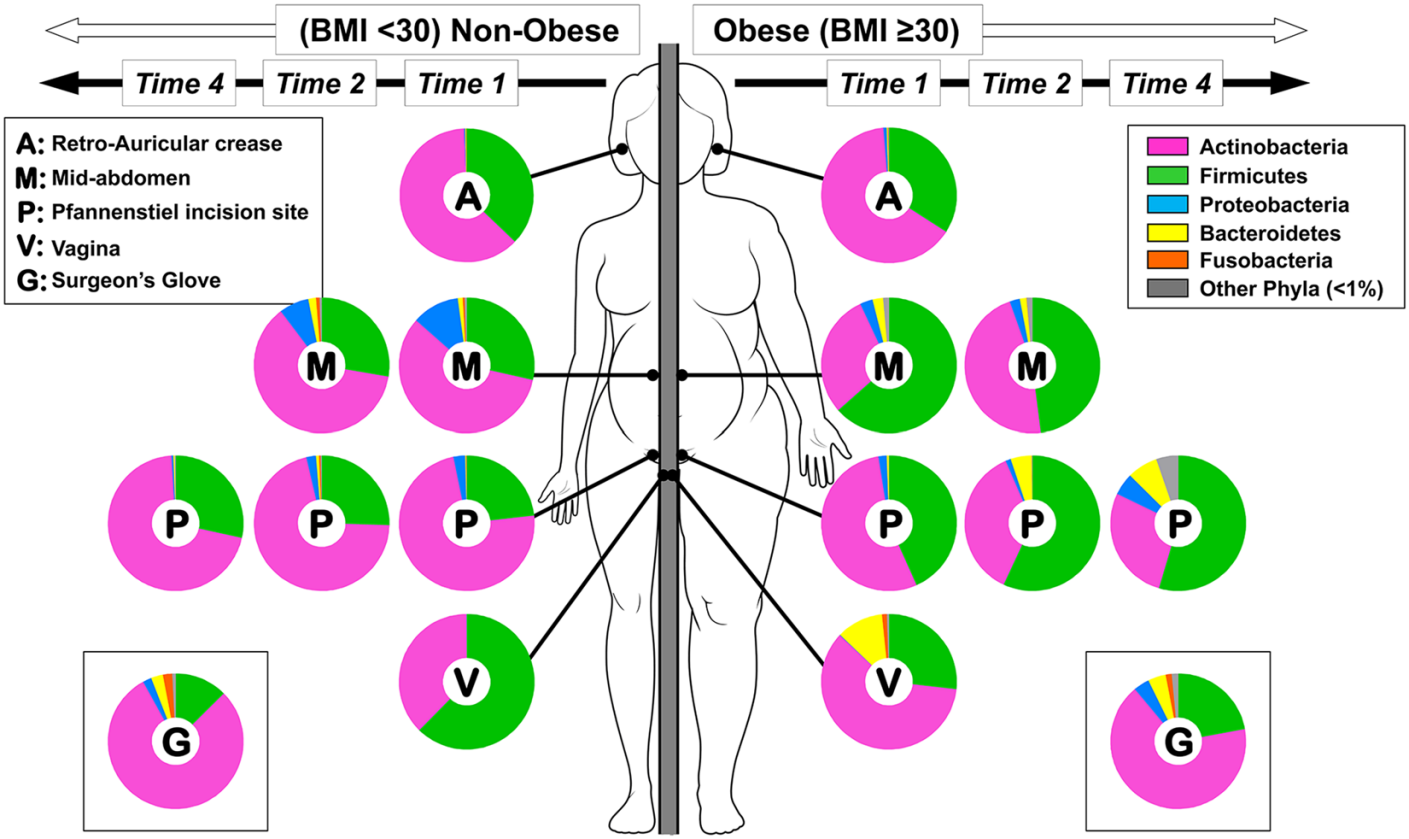
Topographical and temporal changes in microbial communities at the phylum level. As shown the sampled sites had a mixed representation of *Actinobacteria*, *Firmicutes*, *Proteobacteria*, *Bacteroidetes* and *Fusobacterium* with *Tenericutes*, *Synergistetes* contributing <1% to any given sample. *Actinobacteria* and *Firmicutes* represented >80% of bacterial DNA.

Consistent with previous reports^18^, we identified at all skin sites a mixed representation of the phyla *Actinobacteria*, *Firmicutes*, *Proteobacteria*, *Bacteroidetes* and *Fusobacterium* with the remaining phyla (e.g. *Tenericutes*, *Synergistetes*) contributing <1% to any given sample. *Actinobacteria* and *Firmicutes* represented >80% of bacterial DNA (Fig. 3).

#### Time 1

Prior to skin prep, the most striking differences between obese and non-obese groups were observed at mid-abdomen and Pfannenstiel areas. At both these sites, obese women an increased representation of *Firmicutes* and *Bacteroidetes* with proportional decreases in *Actinobacteria*. The vaginal microbiota of obese women was characterized by reduced representation of *Firmicutes* and an increased frequency in *Actinobacteria* and *Bacteroidetes*.

#### Time 2

Surgical scrub was associated with a decreased proportion of *Actinobacteria* and *Proteobacteria*, but with an increased proportion of *Firmicutes*, *Bacteroidetes* and *Fusobacteria*.

#### Time 4

Examining the profile of phyla on the Pfannenstiel incision post-surgery, we noted an increased proportion of *Proteobacteria*, *Bacteroidetes* and of other less common phyla with further decrease in *Actinobacteria*. Post-surgery, in obese women the surgeon’s glove had higher abundance of *Firmicutes*, and *Bacteroidetes*. Additional analyses with interpretation in the context of current knowledge are included in Supplementary Appendix S2.

### Topographical and Temporal Changes in Skin Phylotypes

**Figure 4:**
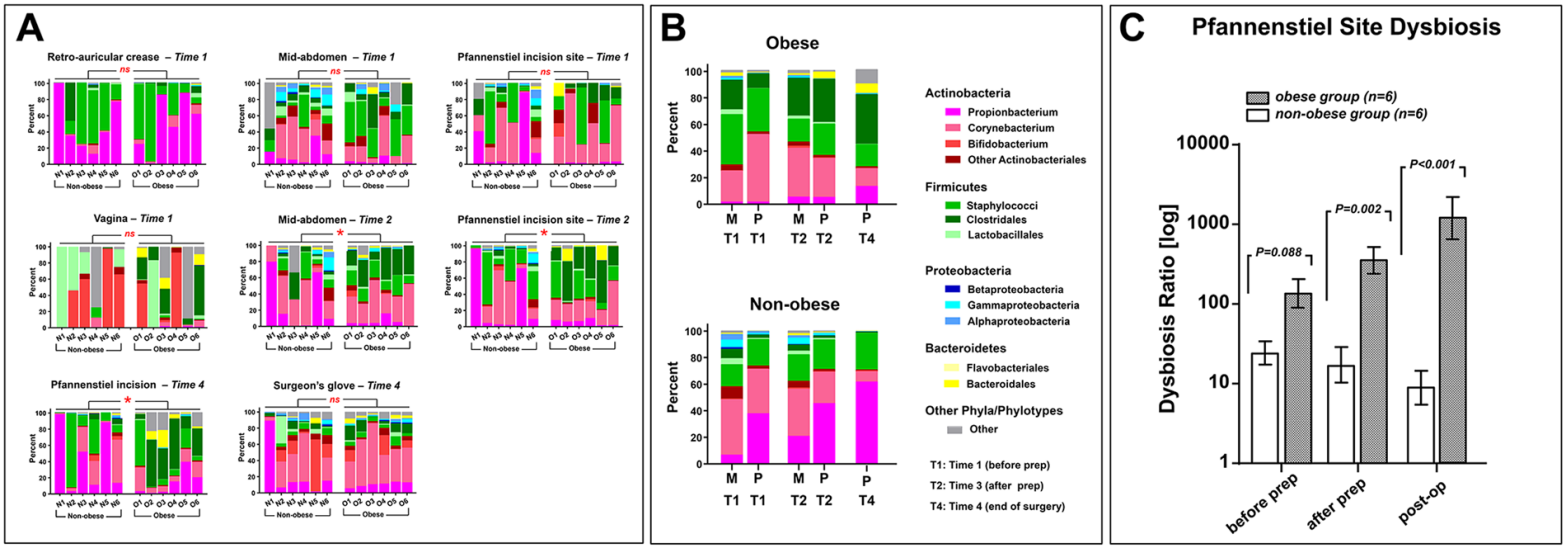
Topographical and temporal changes in skin microbial phylotypes and skin dysbiosis at the Pfannenstiel site during planned Cesarean delivery. Individual (**A**) and average (**B**) changes among obese (n = 6) and non-obese (n = 6) patients at the indicated time points during Cesarean delivery. (**C**) Pfannenstiel site dysbiosis was observed in obese women throughout Cesarean delivery.

The inter-personal variation in microbial community composition displayed as taxonomic phylotypes of Grice and Segre^18^ along with their phyla-level classification is shown in Fig. 4A. After adjustment for inter-personal variation, there were significant differences in microbial community composition between obese and non-obese women at mid-abdomen after prep and at Pfannenstiel site after prep and post-surgery (Supplementary Table S2). The phylotypes accounting for the differences between obese and non-obese women at these sites and times are listed in Supplementary Table S3. Among discriminating phylotypes, *Clostridales* showed consistent enrichment at sites of obese women.

The phylotypes responsible for the differences in community composition before and after skin preparation of obese and non-obese women are presented in Supplementary Table S4 (temporal dissimilarity). At Pfannenstiel site, skin preparation of obese women resulted in increased representation of *Clostridales* (genera *Anaerococus*, *Peptoniphilus*, *Finegoldia*) and *Bacteroidales* (genera *Prevotella*, *Porphyromonas*) with decrease in the *Corynebacterium* and *Staphylococcus*. These changes partially reflected at mid-abdomen. In non-obese women, the phylotype maximally enriched at both Pfannenstiel and mid-abdomen, was *Propionbacteria* (member of *Actinobacteria*). Next, we analyzed the difference in skin microbiota between mid-abdomen and Pfannenstiel (topographical dissimilarity, Supplementary Table S4). After prep, the dissimilarity between Pfannenstiel and mid-abdomen narrowed for obese (before prep: 51.9% vs. after prep: 32.1%), but not for non-obese women (before prep: 57.0% vs. after: 57.9%). This can be further appreciated visually from Fig. 4B.

The dissimilarity in microbiota on the Pfannenstiel incision at the end of CD (*Time 4*) compared to prior to incision (*Time 2*, after prep) was underlined in obese women by an additional increase in representation of *Clostridales (genera Peptoniphilus Anaerococus*, *Finegoldia* and *Ezakiella)* with decrease in *Staphylococci* and *Corynebacteria*. At the end of CD, *Clostridales* became the most abundant incisional phylotype (35.6%). The rare phyla increased in representation were led by *Synergystetes* and were identified only in obese women (genera *Jonquella* and *Pyramidobacter*). In contrast, for non-obese women, the difference after surgery was accounted primarily by further increase in representation of *Propionbacteria* and *Staphylococci* (Supplementary Table S5). An increase in *Propionbacterium* was noted also in obese women but the representation of this phylotype at the end of CD was less than a third than in non-obese.

### Temporal Changes in Skin Dysbiosis at Pfannenstiel Site

We estimated the level of dysbiosis at the surgical site based on the abundance of *Clostridales* and *Bacteroidals* phylotypes relative to skin the commensals *Propionbacteria* and *Staphylococcus*. The dysbiotic ratio between obese and non-obese women becomes significant after skin antisepsis and further increased after surgery (Fig. 4C).

### Changes in Skin Genera and in Alpha-Diversity

Clinically relevant comparisons in alpha-diversity metrices for richness (Fisher’s alpha index), dominance (Berger-Parker *d* index) and diversity (Shannon-Weiner H index) at genera level are presented in Supplementary Fig. S1. In obese women, skin preparation of the Pfannenstiel site resulted in increased species richness and bacterial diversity, but a decreased of the dominance index which also remained lower post-op.

**Figure 5:**
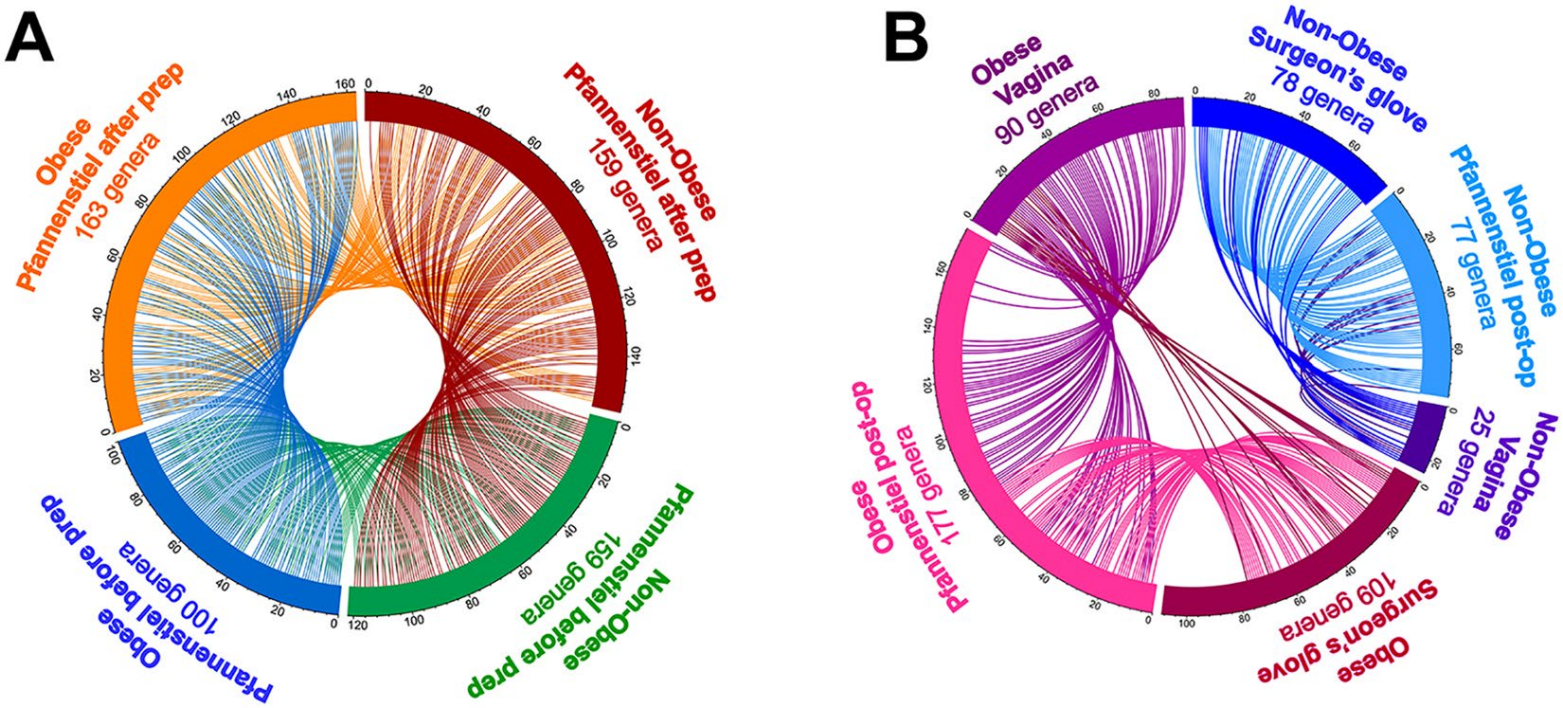
Circos visualization of bacterial genera shared among pre- and post-op Pfannenstiel incision (**A**), and post-op Pfannenstiel site, vagina and surgeon’s glove (**B**) in obese and non-obese women undergoing scheduled Cesarean delivery. After prep, the Pfannenstiel site showed more genera compared to pre-op. The enrichment was higher in obese women. Shared genera were observed between Pfannenstiel incision post-op, vagina and surgeon’s glove, and the extent of overlap was higher for obese women.

The Circos visualization of shared genera at the Pfannenstiel area before and after prep is presented in Fig. 5A. After prep, the Pfanneniel site harbored more genera compared to pre-op. The enrichment in new genera was higher in obese women (82 vs 69 new genera) with only 19 new genera in common implying a unique profile in obese women. The same type of analysis among Pfannenstiel incision post-op, vagina and surgeon’s glove demonstrated shared genera with the extent of overlap higher for obese women (11 genera common to all 3 sites for non-obese vs. 32 for obese women) Fig. 5B. Of sequences matching to genera uniquely shared in obese women, 87% matched to *Clostridales* and *Bacteroidals* phylotypes (leading genera: *Anaerococcus*, *Peptoniphilus*, *Finegoldia*, *Porphyromonas*).

### Identification of biofilms on skin biopsy after surgical antisepsis

Biofilms represent a reservoir of hidden bacteria which can become mobilized upon scrubbing. Of the 17 skin biopsies that were both positive by Gram stain and also had associated keratinized epithelium, 13 (76%) were positive for integration host factor (IHF). IHF is one of two bacterial DNA-binding proteins of the DNABII family that are abundant in biofilms and, along with extracellular bacterial DNA, provide essential structural support to the biofilm^19–21^. Positive staining for IHF was independent of BMI. Representative micrographs of skin biopsies from 4 women are presented in Supplementary Fig. S2.

## Discussion

Compared with non-obese women, obese mothers have significantly higher pre-operative bacterial biomass at the anticipated Pfannenstiel incision site. Skin pre-operative antisepsis with CHG decreases bacterial quantity at the future surgical site to a point where obese and non-obese women begin surgery with similar bacterial loads. Excess bacteria from the Pfannenstiel area of obese woen may have been displaced to the mid-abdomen since this site and surgical scrub showed a significant gain in bacteria immediately following antisepsis. We demonstrated a considerable surge in bacterial DNA load on the surgeon’s glove, and the Pfannenstiel incision at the end of surgery both in obese (2.4-fold) and non-obese (3.3-fold) women. We provided pragmatic evidence that despite strict aseptic techniques and maintenance of a safe environment in the operating room, elective CD is not a sterile procedure. We identified a significant gain in bacterial biomass at the end of surgery in both obese and non-obese women on the Pfannenstiel site which is expected to be essentially sterile. The significance of our findings is supported by previous studies concluding tissue bacterial load has independent predictive value for subsequent infection^22–26^.

The commensal, mutualistic, parasitic and saprophytic relationships established between host’s skin and its inhabiting microorganisms has been reviewed in numerous publications^27–31^. Our findings are in agreement with the notion that skin occlusion favoring an acidic pH and high humidity favors growth of *Firmicutes*, known to be associated with SSI in obese non-pregnant patients^32^. Examination of the phyla on the Pfannenstiel incision post-surgery revealed an increased proportion of *Proteobacteria*, and of less common phyla. Within these, two species (*Jonquella* and *Pyramidobacter* spp.) in phylum *Synergystetes* had the leading representation. *Synergystetes* is a newly classified gram-negative anaerobic phylum identified in periodontal, infections^33^. Interestingly, mice fed a high fat diet reported *Synergistetes* part of the “obese microbiota” with translocation resulting from loss of gut barrier^34^. Thus, we raise the possibility of *Synergystetes* as potential etiologic agent of polymicrobial nature of SSI in obese women. Collectively, our findings imply that obese pregnant women undergoing CD may have an increased risk of SSI, due to anatomical differences from the pannus, which creates a unique moist environment at the anticipated site of surgery. It further suggests that surgical antisepsis which is expected to disrupt skin biofilms may uncover a unique profile of microbiota skin biofilm resulting in increased bacterial richness and diversity, and decreased dominance in obese women. This intervention, in certain patients, may disrupt the equilibrium of the “good” commensal skin bacteria and the “bad” pathogenic bacteria, further increasing risk of infection.

The skin and genital tract are the most influential reservoirs for bacterial contamination^35–37^. Traditionally, minimizing the bioburden of cutaneous microflora on the surgical site prior to surgery or intra-operatively is fundamental for prevention of SSI. Our results suggest marked increased in bacterial DNA at Pfannenstiel incision post-surgery in both groups, questioning the overall sterility of the current surgical technique for CD. The lower uterine segment and the physician’s glove after delivery of the baby’s head demonstrated these sites were rich in bacterial DNA, implying they may be the source of bacterial DNA being deposited on the maternal skin at the end of surgery. Our inference is supported by finding shared common genera between Pfannenstiel incision post-op, vagina and surgeon’s glove, with more overlap in obese women. Recently, the concept and benefits (i.e. asthma, atopic disease) of neonatal vaginal seeding, immediately after CD, received increased attention^38,39^. Our analysis of the shared genera among Pfannenstiel incision, vagina and surgeon’s glove demonstrates this colonization process occurs in the context of the current surgical technique of CD thus implying vaginal seeding is unnecessary. However, routine cleansing the vagina with antiseptic solution, changing the entire surgical team’s gloves and surgical instruments intra-operatively, after delivery of the placenta, would reduce the rate of post-CD wound infections^40,41^.

Biofilms are described as microbial communities that attach to an interface or to each other, and are embed into a matrix of extracellular polymeric substances (EPS) aimed to protect microorganisms from outside perturbations^14^. Biofilms allows for microbial communication, enhanced virulence and breakdown of nutrients aiding microbial succession and development^14^ .Biofilms tolerate antimicrobial properties of the immune system, antiseptics and antibiotics^42,43^. Specifically, the antimicrobial effectiveness of ChloraPrep^TM^ was reduced when challenged by *S*. *epidermidis* and *S*. *aureus* (Firmicutes), when in a biofilm formation^15,16^ .In our study we identified persistence of biofilms after surgical antisepsis. Our literature search suggests most microorganisms identified using metagenomic tools carry the ability to adhere and form biofilms^44^. Overall, by using sequencing technology, we identified insight into the nature of skin microbiome in obese women undergoing CD. To our knowledge there have been no prior studies of the skin microbiota in pregnant women or as this relates to BMI. Our study provides first necessary step before prospective clinical studies of surgical outcome could be effectively designed to underscore the importance of incorporating targeted SSI prophylaxis and practices to disrupt skin biofilms that are not routinely disrupted using the current alcohol-based chlorahexidine solutions.

## Methods

### Patients and Study Design

Using a prospective observational study design, we enrolled 58 consecutive women pregnant with singletons who were scheduled for CD at term (≥37weeks). Indications for CD were: prior CD (n = 51), suspected fetal macrosomia (n = 3), malpresentation (n = 2), history of prior shoulder dystocia (n = 1) and patient preference (n = 1). Clinical signs of labor, membrane rupture, diabetes, positive HIV or hepatitis status, known active infections or antibiotics use during the past 7 days were considered exclusion criteria. All patients received routine prophylactic antibiotics in accordance with the current recommendations^10^ .All women were recruited at The Ohio State University Wexner Medical Center and provided written informed consent. Experimental protocol, data collection, and consent forms were approved by The Ohio State University Human Research Protection Program and all methods were performed in accordance with the relevant guidelines and regulations.

Women were enrolled at admission for surgery. We took a pragmatic approach with respect to pre- and intra-operative skin antisepsis and to the surgical technique for CD. Details about data collection prior to skin preparation before surgery are provided in Supplementary Appendix S1. A schematic of the sampled sites relative to antisepsis and surgical times is provided in Fig. 1. *Time 1 (pre-antisepsis)*, involved swabs from retro-auricular crease (behind the ear), volar forearm, mid-abdomen, anticipated site of Pfannenstiel incision, ChloraPrep^TM^ applicator, vagina, and surgeon’s glove.

At *Time 2 (pre-operative)*, after skin prep and before skin incision we obtained swabs from the same mid-abdomen and Pfannenstiel sites, and ChloraPrep^TM^ applicator after skin prep. *Time 3 (intra-operative)*, after skin incision and prior to opening of the peritoneal cavity involved excision of a small sample of skin from the lower edge of the incision, and collection of samples from lower uterine segment and surgeon’s glove after delivery of the neonate. *Time 4 (post-operative*), after skin closure, and prior to application of the sterile dressing, involved collection of a **s**wab directly from the surface of the incision. To minimize variability, the skin prep and collection of the swabs was performed by a single investigator (KMR). Technical details about sites and sample collection are provided in Supplementary Appendix S1.

### DNA Extraction, Measurement of Bacterial Load and Identification of Bacterial Biofilm

Following collection, all swabs were immediately transported in their sterile containers and stored at −20 °C until processing (2–3 months). DNA was isolated and cleaned up with QIAamp DNA mini-kit (QIAGEN). 16 S rRNA gene was amplified by RT-PCR using specific primers and probe sets. Skin samples from the Pfannenstiel site were fixed in formalin and cryo-embedded. Details on laboratory techniques are provided in Supplementary Appendix S1.

### Sequencing of 16 s RNA Genes and Microbial Community Analysis

DNA samples were submitted for metagenomics analysis. The amplified gene segments where sequenced using the ABI Prism 3100xl genetic analyzer (Applied Biosystems), a 16-capillary instrument using fluorescence based electrophoresis technology for DNA sequencing and fragment analysis at The Ohio State University DNA core facility (Wooster, OH). 16 S metagenomics analysis was carried out as per Schloss *et al*.^45^ and Kozich *et al*.^46^ using the most recently available version of mothur (http://www.mothur.org, ver1.40.0).

### Data Processing and Statistical Analyses

Details about processing of the sequence reads, analysis of the genera as described by Grice and Segre^18^, and additional statistical analyses are provided in Supplementary Appendix S1.

### Data Availability

The datasets generated during and/or analyzed during the current study are available from the corresponding author on reasonable request.

## Electronic supplementary material

Supplementary Information
