# Supplementary material for: Skin Microbiota in Obese Women at Risk for Surgical Site Infection After Cesarean Delivery
Source: Sci Rep. 2018 Jun 8;8:8756. doi: 10.1038/s41598-018-27134-5 (PMC5993816; doi:10.1038/s41598-018-27134-5)
Supplement: Supplementary file 1 — Supplementary Information [file 41598_2018_27134_MOESM1_ESM.docx]

**Supplementary Online Content**

**Skin microbiota in obese women at risk for surgical site infection after cesarean delivery**

Kara M. Rood^1^, Irina A. Buhimschi^2,3^, Joseph A. Jurcisek^5^,

Taryn L. Summerfield^1^, Guomao Zhao^2^, William E. Ackerman 4^th1^, R. Wolfgang Rumpf^4^, Weiwei Wang^2^, Stephen F. Thung^1^, Lauren O. Bakaletz^5^, and Catalin S. Buhimschi^1^

**Supplementary Appendix S1.** Supplementary Methods

**Supplementary Appendix S2.** Topographical and Temporal Changes in Microbial Communities at Phylum Level

**Supplementary Table S1.** Demographic, clinical, and surgical outcome characteristics of the women undergoing scheduled cesarean delivery at term

**Supplementary Table S2.** Microbial community similarity and dissimilarity results between obese and non-obese women accounting for interpersonal variation in microbial phylotypes

**Supplementary Table S3.** Phylotypes accounting for dissimilarity between obese and non-obese women at clinically relevant skin sites

**Supplementary Table S4.** Phylotypes accounting for dissimilarity after surgical skin preparation (temporal) at mid-abdomen and Pfannenstiel sites (topographical)

**Supplementary Table S5.** Phylotypes accounting for temporal dissimilarity between Pfannenstiel site post-surgery with Pfannenstiel site pre-surgery

**Supplementary Figure S1.** Clinically relevant comparisons in alpha-diversity metrices for richness (Fisher's alpha index), dominance (Berger-Parker *d* index) and diversity (Shannon-Weiner H index) at genera level

**Supplementary Figure S2.** Representative images of residual bacterial biofilms on *stratum corneum* of the Pfannenstiel skin incision after preoperative surgical antisepsis

**Supplementary References**

This supplementary material has been provided by the authors to give readers additional information about their work.

**Supplementary Appendix S1.**

**Supplementary Methods**

Skin preparation prior to surgery. After enrollment, women were questioned about their demographic and medical history followed by information on current pregnancy. The time of last shower was recorded along with any use of skin lotions and antiseptics on the abdominal skin area within the last 24 hours. At our institution all women scheduled for CD are provided with two bottles of chlorhexidine gluconate (CHG) soap (Ecolab, St. Paul, MN) and instructed to cleanse the abdomen with it while showering, the night before and the morning of their scheduled surgery. In addition, on the day of surgery upon presentation to pre-operative area, each woman undergoes cleansing of the abdominal skin area by trained nursing personnel using two pre-operative skin-friendly Sage 2% CHG cloths (Sage Products, Cary IL).

Once in the operating room, a standard surgical skin antisepsis (skin prep) with ChloraPrep^TM^ (2% CHG in 70% isopropyl alcohol, Becton Dickinson, Franklin Lakes NJ) was performed on all cases. Moving from side to side, the skin prep started in the anticipated site of the Pfannenstiel incision to cover an area that extended cranially from one inch below the xyphoid process to mid-thigh caudally. Two ChloraPrep^TM^ applicators were used in all cases and a 3-minute drying time occurred prior to application of sterile surgical drape.

Sampled sites relative to antisepsis and surgical times. Before prep (*Time 1, pre-antisepsis*), we obtained swabs from retro-auricular crease (behind the ear), volar forearm (midway between the wrist and antecubital fossa), mid-abdomen (midline half-way between umbilicus and symphysis pubis) and from the anticipated site of Pfannenstiel incision (midline 2-3 cm above the symphysis pubis).

After skin prep and before skin incision (*Time 2, pre-operative*) we obtained another set of swabs from the same mid-abdomen and the Pfannenstiel sites. To ensure any observed skin microbial profiles are not impacted through external contamination, we collected a swab from the ChloraPrep^TM^ applicator before (*Time 1*) and after skin prep (*Time 2*). After skin incision but prior to entering the abdominal cavity (*Time 3, intra-operative*), a small sample of skin was excised from the lower edge of the incision. Lastly, at the end of the procedure after skin closure, and just prior to application of sterile dressing (*Time 4, post-operative*) we obtained an additional swab directly from the surface of the incision.

Because a preliminary analysis of the first 8 cases found strikingly higher bacterial loads at the Pfannenstiel site at *Time 4* compared to *Time 2,* four additional swabs were performed in the rest of the cases (n=50) to track potential sources of the added bacterial DNA: from the posterior fornix of the vagina (at *Time 1*), of the inside of the anterior wall of the lower uterine segment (at *Time 3*, after delivery of the baby and placenta) and of the surgeon’s glove before (at *Time 1*) and after delivery of the fetal presenting part (at *Time 3*). In total, each case generated 9-13 swab specimens for a total of 772 swabs collected during this study. To minimize variability, the skin prep and collection of the swabs was performed by a single investigator (KMR).

Technical details for collection of swab specimens. All specimens were collected using sterile gloves and sterile Catch-All™ sample collection swabs (Epicentre Biotechnologies, Madison WI) according to the Manual of Procedures for the Human Microbiome Project.^^[[1]](#endnote-1)^^

For skin surfaces, the swab was pre-moistened in sterile saline solution then rubbed back and forth ~50 times along the sampling area, while applying firm pressure for 30 seconds. Swabs from the Chloraprep^TM^ applicator and from the surgeon's glove were obtained in similar fashion. Swabs from the lower uterine segment and the vagina were wiped against the organ wall for 30 seconds.

DNA Extraction and Measurement of Bacterial Load. Following collection, all swabs were immediately transported in their sterile containers and stored at -20ºC until processing (2-3 months). DNA was isolated with bead-beating method and cleaned up with QIAamp DNA mini-kit (QIAGEN). The quantification and quality check was performed with Thermo Scientific NanoDrop 2000/2000c Spectrophotometer (Thermo Fisher Scientific, Waltham MA). All procedures were carried out under sterile conditions.

16S rRNA gene was amplified by RT-PCR. The following 16S rDNA primers and probe set (Integrated DNA Technologies, Coralville Iowa) were used: forward primer, 5’-TCC TAC GGG AGG CAG CAG T-3’ (T_m_, 60.8 ^o^C), reverse primer, 5’-GGA CTA CCA GGG TAT CTA ATC CTG TT-3’(T_m_, 57.2 ^o^C), and 16S universal probe, 5’-/56-FAM/CGT ATT ACC GCG GCT GGC AC/36-TAMSp/-3 (Tm, 65.6 oC).^^[[2]](#endnote-2)^^

The following TaqMan Gene Expression Assay (Applied Biosystems, Foster City, CA) was used as a housekeeping gene: Hs03882242_s1 (GPI). A standard bacterial stock of *Porphyromonas gingivalis* was used (ATCC, Manassas, VA). Each 20 µl reaction consisted of 1 µl DNA, 1 µl of 16S Primers/probe set (20x) or TaqMan Gene Expression Assay, 10 µl TaqMan Fast Advanced Master Mix (Applied Biosystems, Foster City, CA), and 8 µl of nuclease free water. Amplifications were performed on the StepOnePlus Real-Time PCR System (Applied Biosystems, Foster City, CA).

Identification of Bacterial Biofilm in Skin Biopsies. Skin samples from the Pfannenstiel site were fixed in formalin and embedded in paraffin. Ten micron serial sections were cut using a Leica microtome, adhered to glass slides (Mercedes Medical, Sarasota, FL), and stored at room temperature. Slides were deparaffinized, hydrated through graded alcohols to water, then equilibrated in buffer (0.05 M Tris-HCl, 0.15 M NaCl, and 0.05% Tween 20, pH 7.4). Sections were then blocked with Image-iT FX signal enhancer (Molecular Probes, Eugene, OR) and with Background Sniper (BioCare Medical, Concord, CA) as per manufacturer's instructions. Sections were then incubated overnight at 4°C with a 1:200 dilution of polyclonal rabbit anti-Integration Host Factor (IHF, a member of DNABII family of proteins which contributes to structural integrity of biofilms) as previously described.^i^ Slides were then rinsed and incubated with goat anti-rabbit IgG conjugated to Alexa eFluor 594 (Invitrogen, Eugene, OR) for 30 min. at room temperature. As counterstain for DNA, sections were incubated with 4′,6-diamidino-2-phenylindole (DAPI) and cover-slipped using ProLong Gold Anti-fade Reagent (Molecular Probes, Eugene, OR). Use of naive rabbit serum in place of immune serum and use of secondary antibody alone served as negative control preparations. Adjacent sections were stained with hematoxylin-eosin (H&E) and Gram stain using the protocol modified by Becerra et al.^[[3]](#endnote-3)^ Sections were viewed with a Zeiss Axiovert 200M inverted microscope (Carl Zeiss, Thornwood, NY).

Sequencing of 16s RNA Genes and Microbial Community Analysis. Next-Gen sequence reads were generated with primers 47F and 543R and were screened to remove ambiguous and non-unique sequences with a 540bp size cutoff. The resulting sequences were aligned against the most recent version of SILVA rRNA database (http://www.arb-silva.de, ver128)^[[4]](#endnote-4)^ and filtered for uniqueness. Pre-clustering was performed with *diffs=2* to ensure accurate clustering of orthologous sequences despite sequencing-induced “mutations” or errors. Chimeric sequences were removed using VSEARCH 2.44 ^[[5]](#endnote-5)^ and sequences were classified using Silva v128 taxonomy ^[[6]](#endnote-6)^ with a cutoff of 0.80. Non-prokaryotic sequences were removed prior to clustering with a cutoff of 0.15, resulting in a final data set of unique prokaryotic operating taxonomic units (OTUs). A custom R script ^[[7]](#endnote-7)^ was used to combine the contents of the mothur-generated *shared* and *taxonomy* files to create a taxonomic representation of bacterial counts per sample collapsed at the genus level. For analyses purposes genera were either grouped by phyla or by the taxonomic categories (phylotypes) previously espoused by Grice and Segre to define relationships between skin site types (sebaceous, moist, dry) and their respective microbiome profiles.^[[8]](#endnote-8)^ Multinomial Goodness-of-Fit tests were used to compare differences in microbial profiles at phylum level. Community Analysis Package ver. 5 (CAP5) and Species Diversity & Richness ver.4.1.2 (SDRIV, Pisces Conservation) statistical software were used to analyze composition of microbial communities at the sampled sites and times. Intra- and inter-personal variation in microbial community membership to Greice and Segre phylotypes was calculated from similarity and dissimilarity indices using CAP5's ANOSIM package^[[9]](#endnote-9)^. The SIMPER package based on Bray Curtis distances was used to extract phylotype contributions to dissimilarity for clinically relevant comparisons. Indices of alpha diversity (richness, dominance were used to characterize microbial communities at genus level.

Additional Statistical Analyses. We performed an *a priory* sample size calculation based on the data reported previously by Grice and Segre that identified differences in microbiome profiles between different skin types.^8^ Ten human subjects were determined sufficient to detect differences with the use of 16S rRNA sequencing. Because there was no prior experience with the magnitude of the expected difference at the Pfannenstiel site between obese and non-obese women, we recruited a larger number of subjects (n=58) to facilitate finer comparisons and account for confounders and possible effect modifiers.

Normality testing was performed using the Shapiro-Wilk test. Bacterial load data was analyzed after logarithmic transformation. Comparisons among groups and time points employed Student t-tests, 1-way or 2-way ANOVA (parametric) or Mann-Whitney tests (non-parametric) and chi square using Sigma Stat ver. 2.03 (SPSS Inc., Chicago, IL) and MedCalc (Broekstraat, Belgium). Spearman correlations were used to measure co-linearity between variables. Stepwise multivariate linear regression was used to model potential interactions between variables. A *P* value of <0.05 or less was consider statistically significant.

**Supplementary Appendix S2.**

**Topographical and Temporal Changes in Microbial Communities at Phylum Level**

Although to our knowledge there has been no prior study of microbiota on the skin of pregnant women, there is general consensus that microbial communities that inhabit the human skin mainly fall into four phyla: *Firmicutes*, *Bacteroidetes*, *Proteobacteria* and *Actinobacteria,* in highly variable proportions depending on anatomical location, endogenous host factors and exogenous environmental factors.^29,^^[[10]](#endnote-10),^^[[11]](#endnote-11)^ A marked difference in representation of phyla has been described between moist, sweaty hairless skin of the antecubital fossa and the dry, less sweaty, more haired skin of the volar forearm.^^[[12]](#endnote-12)^^ Partially occluded areas of skin, such as groin, and axillary vault have higher temperature and humidity that encourage predominant growth of *Corynebacterium* spp. (phylum *Actinobacteria*), in contrast to sebaceous areas, where *Propionibacteria* spp. (phylum *Actinobacteria*) and *Staphylococci* spp. (phylum *Firmicutes*) predominate. Dry areas are known to favor greater prevalence of *β-Proteobacteria* (phylum *Proteobacteria*) and *Flavobacteriale* spp. (phylum *Bacteroidetes*).^29,^^[[13]](#endnote-13),^^[[14]](#endnote-14)^

In the current study we made the following observations:

*Time 1.* Prior to skin prep, the most striking differences between obese and non-obese groups were observed at mid-abdomen and Pfannenstiel areas. At both these sites, obese women had an increased representation of *Firmicutes* (mid-abdomen: non-obese: 28% vs. obese: 63%; Pfannenstiel: non-obese: 23% vs obese 43%; *P*<0.001 for both) and *Bacteroidetes* (mid-abdomen: non-obese: 1.2% vs. obese: 2.5%; Pfannenstiel: non-obese: 0.2% vs obese 0.5%; *P*<0.001 for both) with proportional decreases in *Actinobacteria.* The vaginal microbiota of obese women was characterized by reduced representation of *Firmicutes* (non-obese: 62% vs. obese: 27%, *P*<0.001), and an increased frequency in *Actinobacteria* (non-obese: 38% vs. obese: 60%, *P*<0.001) and *Bacteroidetes* (non-obese: 0% vs. obese: 11%, *P*<0.001).

*Time 2.* In addition to the previously noted reduction in bacterial load, surgical prep of the Pfannenstiel site of obese women changed the relative phyla proportion. Specifically, surgical scrub was associated with a decreased proportion of *Actinobacteria* (before prep 54% vs. after 37%, *P*<0.001) and *Proteobacteria* (before prep 2% vs. after 1.3%, *P*<0.001), but with an increased proportion of *Firmicutes* (before prep 43% vs. after 57%, *P*<0.001), *Bacteroidetes* (before 0.5% vs. after 5%, *P*<0.001) and *Fusobacteria* (before prep 0.001% vs. after, 0.020%, *P*<0.001).

*Time 4.* Examining the profile of phyla on the Pfannenstiel incision post-surgery, we noted an increased proportion of *Proteobacteria* (obese post-surgery 5.3% vs. after prep 1.3%, *P*<0.001), *Bacteroidetes* (obese post-surgery 7.3% vs. after prep 5.0%, *P*<0.001) and of other less common phyla (obese post-surgery 5.3% vs. after prep 0.1%, *P*<0.001) with further decrease in *Actinobacteria* (obese post-surgery 28% vs. after prep 37%, *P*<0.001). Post-surgery, in obese women the surgeon's glove had higher abundance of *Firmicutes (*22% vs 13% for non-obese, *P*<0.001), *Bacteroidetes (*4.3% vs. 2.2% for non-obese, *P*<0.001) and uncommon phyla (1.6% vs. 0.8% for non-obese, *P*<0.001).

**Supplementary Table S1.**

**Demographic, clinical, and surgical outcome characteristics of the women undergoing scheduled cesarean delivery at term**

| **Variables** | **BMI <30**  **(Non-Obese)**  **n = 27** | **BMI ≥30**  **(Obese)**  **n = 31** | ***P* value** |
| --- | --- | --- | --- |
| ***Maternal characteristics at enrollment*** | | | |
| Age, *years*, † | 27 [22 – 37] | 26 [21 – 38] | 0.616 |
| Non-Caucasian race ‡ | 11 (41) | 11 (36) | 0.888 |
| Gravity † | 3 [2 – 3] | 3 [2 – 4] | 0.608 |
| Parity † | 1 [1 – 2] | 1 [1 – 2] | 0.979 |
| Gestational age, *weeks* † | 39 [39 – 39] | 39 [38 – 39] | 0.387 |
| Pre-pregnancy BMI † | 22.9 [20.9 – 24.7] | 31.3 [28.1 – 42.3] | <0.001 |
| BMI at delivery † | 28.6 [26.9 – 29.5] | 37.6 [34.2 – 43.6] | <0.001 |
| Smoker ‡ | 1 (4) | 5 (16) | 0.367 |
| Positive GBS status ‡ | 7 (26) | 9 (29) | 0.768 |
| Prior abdominal surgery ‡ | 25 (93) | 27 (81) | 0.276 |
| Number of prior Cesarean sections ‡  0  1  2  3 | 2 (7)  18 (67)  4 (15)  3 (11) | 5 (16)  14 (45)  9 (29)  3 (10) | 0.327 |
| History of surgical wound infection ‡ | 2 (7) | 2 (6) | 1.000 |
| Panniculus grade ‡  grade 1  grade 2  grade 3 | 27 (100)  0 (0)  0 (0) | 14 (45)  12 (39)  5 (16) | <0.001 |
| Time from last shower, *hours* † | 3 [3 – 4] | 4 [3 – 6] | 0.011 |
| Home body lotion within 24 hours ‡ | 1 (4) | 1 (3) | 0.921 |
| Home CHG soap use within 24 hours ‡ | 24 (89) | 21 (68) | 0.535 |
| ***Surgery characteristics*** | | | |
| Depth of subcutaneous tissue, *cm* † | 1 [1 – 1] | 3 [2 – 4] | <0.001* |
| Length of surgery, *minutes* † | 57 [46 – 67] | 57 [46 – 74] | 0.517 |
| Staples incision closure ‡ | 0 (0) | 12 (39) | <0.001* |
| Estimated blood loss, *mL* † | 700 [500 – 700] | 703 [700 – 900] | 0.004 |
| Silver dressing applied ‡ | 4 (15) | 5 (16) | 1.000 |
| Wound vacuum applied ‡ | 0 (0) | 1 (3) | 1.000 |
| Surgical site infection ‡ | 2 (7) | 7 (22) | 0.111 |

† Data presented as median [interquartile range] and analyzed by Kruskal-Wallis ANOVA on Ranks.

‡ Data presented as n (%) and analyzed by Chi square tests.

*Abbreviations:* *BMI, body mass index; CHG, chlorhexidine gluconate.*

The depth of the subcutaneous tissue and estimated blood loss were higher for obese women. The skin incision was more frequently closed with staples than with subcutaneous suture in the obese group. Within 14 days after Cesarean, 15% (9/58) of enrolled women developed an SSI (BMI<30, n=2 and BMI≥30, n=7, *P*=0.111).

**Supplementary Table S2.**

**Microbial community similarity and dissimilarity between obese and non-obese women accounting for interpersonal variation in microbial phylotypes**

| Site | Time | Within group  average  similarity* | | Between groups  average  dissimilarity* | r † | *P* value † |
| --- | --- | --- | --- | --- | --- | --- |
|  |  | Non-Obese | Obese | Obese vs. Non-Obese |  |  |
| Retroauricular | Time 1 | 49.1 | 49.9 | 43.3 | -0.128 | 0.933 |
| Mid-Abdomen | Time 1 | 46.5 | 55.8 | 54.1 | 0.194 | 0.073 |
| Pfannenstiel | Time 1 | 32.6 | 47.5 | 59.5 | -0.019 | 0.479 |
| Vagina | Time 1 | 34.6 | 24.3 | 74.6 | 0.104 | 0.174 |
| **Mid-Abdomen** | **Time 2** | 38.3 | 71.1 | 51.2 | 0.246 | **0.023** |
| **Pfannenstiel** | **Time 2** | 36.6 | 65.4 | 61.3 | 0.311 | **0.003** |
| **Pfannenstiel** | **Time 4** | 38.0 | 48.9 | 69.4 | 0.335 | **0.021** |
| Surgeon's glove | Time 4 | 39.7 | 69.9 | 46.7 | 0.043 | 0.244 |

* based on SIMPER analysis

† based on ANOSIM analysis. Bold font, *P*<0.05

**Supplementary Table S3.**

**Phylotypes accounting for dissimilarity between obese and non-obese women at clinically relevant skin sites**

| Phylotype | Average  dissimilarity (%) | Average abundance | | Contribution to dissimilarity | | Enrichment  (fold change)* |
| --- | --- | --- | --- | --- | --- | --- |
|  |  | Non-Obese | Obese | Individual % | Cumulative % |  |
| **Mid-Abdomen ‒ Time 2** | | | | | | |
| *Propionbacteria*  ***Clostridales***  *Corynebacteria*  *Staphylococci* | 51.2 | 28.8  8.2  30.5  10.5 | 5.6  28.1  36.3  16.7 | 26.4  21.6  17.2  11.7 | 26.4  48.0  65.2  76.9 | -5.1  **+3.4**  +1.2  +1.6 |
| **Pfannenstiel incision site ‒ Time 2** | | | | | | |
| ***Clostridales***  *Propionbacteria*  *Corynebacteria*  *Staphylococci*  ***Bacteroidales*** | 61.3 | 2.4  30.6  26.4  27.4  0.8 | 34.6  5.3  27.7  21.1  6.2 | 26.3  23.3  18.8  17.4  4.8 | 26.3  49.6  68.4  85.8  90.5 | **+14.4**  -5.8  +1.1  -1.3  **+7.8** |
| **Pfannenstiel incision ‒ Time 4** | | | | | | |
| *Propionbacteria*  ***Clostridales***  *Staphylococci*  *Corynebacteria* | 69.4 | 43.7  2.4  27.7  20.0 | 13.0  35.6  16.7  13.7 | 27.8  23.9  19.3  12.7 | 27.8  51.7  71.0  83.7 | -3.3  **+14.8**  -1.7  -1.5 |

SIMPER analysis based on Bray Curtis distances, n=6 women in each group.

* Positive leading sign indicates increase in phylotype representation after surgical skin prep. A negative sign indicates a decrease. Phylotypes enriched >2fold are shown in bold font.

**Supplementary Table S4.**

**Phylotypes accounting for dissimilarity after surgical skin preparation (temporal) at mid-abdomen and Pfannenstiel sites (topographical)**

| Phylotype | Average  dissimilarity (%) | Average abundance | | Contribution to dissimilarity | | | Enrichment  (fold change)* |
| --- | --- | --- | --- | --- | --- | --- | --- |
|  |  |  |  | Individual % | | Cumulative % |  |
| **TEMPORAL DISSIMILARITY** | | | | | | | |
| **Pfannenstiel Site ‒ Obese** | | | | | | | |
| *Staphylococci*  *Corynebacteria*  ***Clostridales***  Other *Actinobacteriales*  ***Bacteroidales*** | 49.7 | Time 1 | Time 2 | 25.4  25.2  23.9  7.7  6.8 | | 25.4  50.6  74.5  82.2  89.1 | -1.2  -1.7  **+3.0**  -4.8  **+2.1** |
|  |  | 24.5  47.2  11.5  8.2  3.0 | 21.1  27.8  34.6  1.7  6.2 |  |  |  |  |
| **Mid-Abdomen ‒ Obese** | | | | | | | |
| *Staphylococci*  *Corynebacteria*  *Clostridales*  Other_Phyla/Phylotypes | 44.1 | Time 1 | Time 2 | 25.1  22.3  19.6  7.5 | | 25.1  47.4  67.1  74.5 | -2.1  +1.6  +1.7  -2.2 |
|  |  | 34.5  22.5  16.7  6.0 | 16.8  36.3  28.1  2.7 |  |  |  |  |
| **Pfannenstiel Site ‒ Non-Obese** | | | | | | | |
| *Propionbacteria*  *Staphylococci*  *Corynebacteria* | 57.9 | Time 1 | Time 2 | 31.0  22.8  22.5 | | 31.0  53.8  76.3 | +1.3  +1.2  -1.1 |
|  |  | 24.4  22.2  28.8 | 30.6  27.4  26.4 |  |  |  |  |
| **Mid-Abdomen ‒ Non-Obese** | | | | | | | |
| ***Propionbacteria***  *Corynebacteria*  Other  *Staphylococci*  *Clostridales* | 54.2 | Time 1 | Time 2 | 25.2  18.8  12.9  11.6  7.5 | | 25.2  44.0  56.9  68.5  76.0 | **+2.4**  +1.1  -1.6  -1.2  +1.1 |
|  |  | 12.0  28.7  11.7  12.3  7.3 | 28.8  30.5  7.4  10.5  8.2 |  |  |  |  |
| **TOPOGRAPHICAL DISSIMILARITY** | | | | | | | |
| **Time 1 ‒ Obese** | | | | | | | |
| ***Corynebacteria***  *Staphylococci*  *Clostridales*  Other *Actinobacteriales* | 51.9 | Mid-Abdomen | Pfannenstiel | 28.7  27.9  12.3  8.1 | | 28.7  56.6  68.9  77.0 | **+2.1**  -1.5  -1.5  +1.5 |
|  |  | 22.5  36.4  16.7  5.3 | 47.2  24.5  11.5  8.2 |  |  |  |  |
| **Time 2 ‒ Obese** | | | | | | | |
| *Clostridales*  *Corynebacteria*  *Staphylococci*  ***Bacteroidales*** | 32.1 | Mid-Abdomen | Pfannenstiel | 23.6  21.1  21.2  9.0 | | 23.6  44.7  65.9  74.9 | +1.2  -1.3  +1.3  **+4.4** |
|  |  | 28.8  36.3  16.7  1.4 | 34.6  27.7  21.1  6.2 |  |  |  |  |
| **Time 1 ‒ Non-Obese** | | | | | | | |
| ***Propionbacteria***  *Corynebacteria*  *Staphylococci*  Other_Phyla/Phylotypes  Other *Actinobacteriales* | 57.0 | Mid-Abdomen | Pfannenstiel | 21.1  20.3  18.7  11.2  6.9 | | 21.1  41.4  60.1  71.3  78.2 | **+2.0**  +1.0  +1.8  -2.7  -1.9 |
|  |  | 12.1  28.7  12.4  11.7  8.2 | 24.4  28.8  22.2  4.3  4.4 |  |  |  |  |
| **Time 2 ‒ Non-Obese** | | | | | | | |
| *Propionbacteria*  *Staphylococci*  *Corynebacteria* | 57.9 | Mid-Abdomen | Pfannenstiel | 31.0  22.8  22.5 | 31.0  53.8  76.3 | | +1.3  +1.2  -1.1 |
|  |  | 24.4  22.2  28.8 | 30.6  27.4  26.4 |  |  |  |  |

SIMPER analysis based on Bray Curtis distances, n=6 women in each group. Phenotypes accounting together for at least 75% dissimilarity are shown. *A positive sign indicates increase in phylotype representation after prep (Time 2) compared to before prep (Time 1) or increased representation at Pfannenstiel compared to mid-abdomen. A negative leading sign indicates a decrease in representation. Phylotypes enriched >2fold are shown in bold font.

**Supplementary Table S5.**

**Phylotypes accounting for temporal dissimilarity between Pfannenstiel site post-surgery with Pfannenstiel site pre-surgery**

| Phylotype | Average  dissimilarity (%) | Average abundance | | Contribution to dissimilarity | | Enrichment at  (fold change)* |
| --- | --- | --- | --- | --- | --- | --- |
|  |  |  |  | Individual % | Cumulative % |  |
| **Obese** | | | | | | |
| *Clostridales*  *Staphylococci*  *Corynebacteria*  ***Propionbacteria***  ***Other Phyla/phylotypes*** | 44.7 | Pfannenstiel Time 2 | Pfannenstiel Time 4 | 23.0  22.1  17.7  13.0  11.7 | 23.0  45.1  62.8  75.8  87.6 | +1.0  -1.3  -2.0  **+2.5**  **+8.7** |
|  |  | 34.6  21.1  27.7  5.3  1.3 | 35.6  16.7  13.7  13.0  11.3 |  |  |  |
| **Non-Obese** | | | | | | |
| *Propionbacteria*  *Staphylococci*  *Corynebacteria* | 56.8 | Pfannenstiel Time 2 | Pfannenstiel Time 4 | 37.4  25.7  22.3 | 37.4  63.1  85.4 | +1.4  +1.0  -1.3 |
|  |  | 30.6  27.4  26.4 | 43.7  27.7  20.0 |  |  |  |

SIMPER analysis based on Bray Curtis distances, n=6 women in each group. Phylotypes accounting together for at least 75% dissimilarity are shown.

* The positive leading sign indicates an increase in phylotype representation on Pfannenstiel incision post-surgery (Time 4, prior to application of wound dressing) relative to pre-surgery (Time 2, after prep). A negative leading sign indicates a decrease in representation. Phylotypes enriched >2fold are shown in bold font.

**Supplementary Figure S1.**

**Clinically relevant comparisons in alpha-diversity metrices for richness (Fisher's alpha index), dominance (Berger-Parker *d* index) and diversity (Shannon-Weiner H index) at genera level**


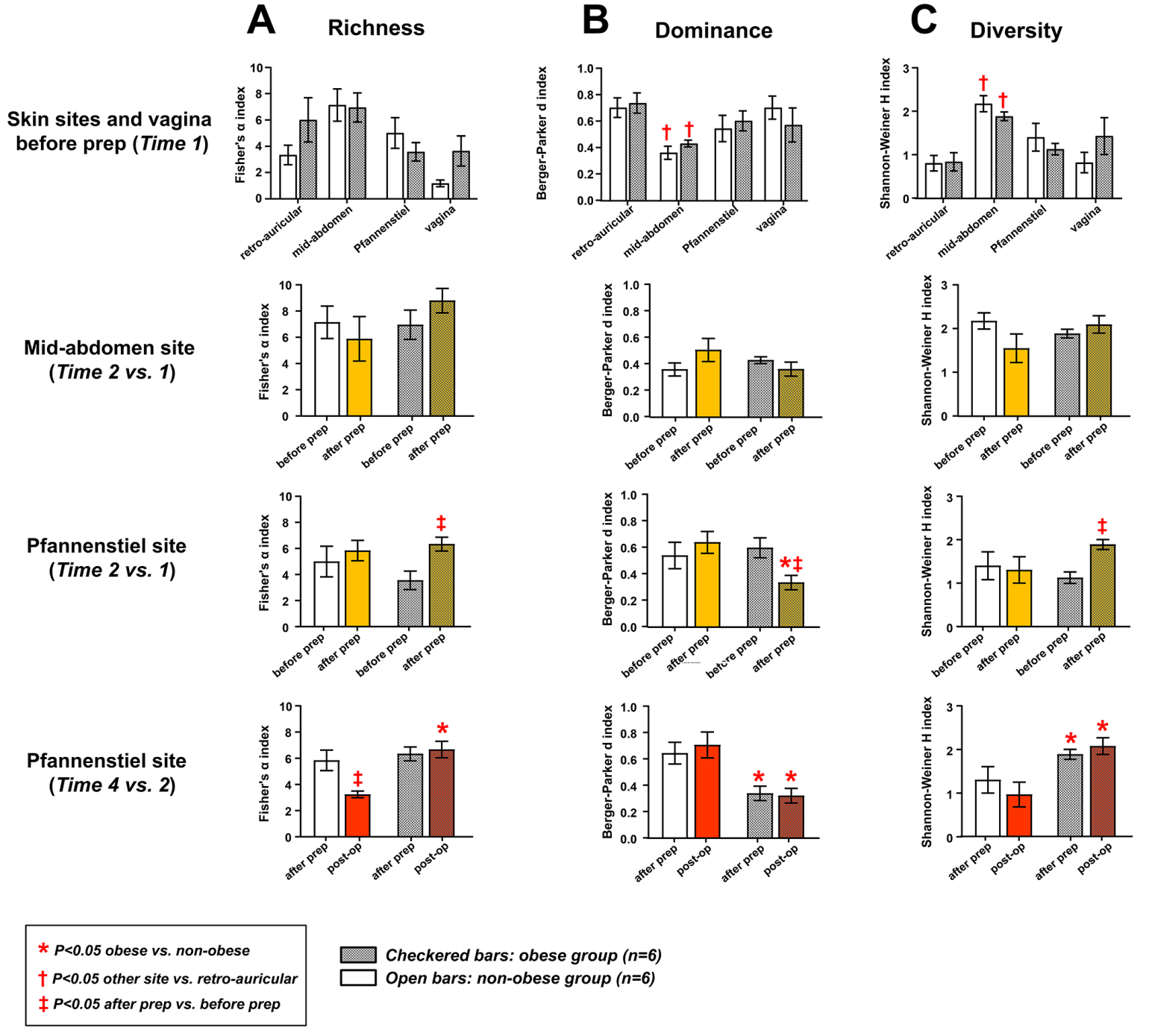


*Richness.* The vaginal site had the lowest richness (98 genera) with no significant differences between obese and non-obese women (Fig. S1A). The highest number of genera was found at mid-abdomen (253 genera). In obese women, species richness increased at Pfannenstiel site after prep (*P*=0.029 vs. before prep). Conversely, non-obese women had lower richness at Pfannenstiel site after surgery (*P*=0.005 vs. after prep).

*Dominance.* In both groups, the highest dominance index was seen retro-auricular (*P*<0.05), and mid-abdomen had the lowest dominance (*P*<0.05) (Fig. S1B). At Pfannenstiel site, skin preparation in obese women decreased the dominance index (*P*=0.030 vs. before prep) which remained lower than for non-obese women both after prep (*P*=0.014) and post-op (*P*=0.003).

*Diversity.* For both obese and non-obese women the highest diversity was observed mid-abdomen (Fig. S1C). Skin preparation at the Pfannenstiel site in obese women increased bacterial diversity (*P*=0.034 vs. before prep) which remained higher than for non-obese women after prep (*P*=0.010) and post-op (*P*=0.002).

**Supplementary Figure S2.**

**Representative images of residual bacterial biofilms on *stratum corneum* of the Pfannenstiel skin incision after preoperative surgical antisepsis**

**Top row (A-D):** Representative images from ten micron thick sections stained with Gram stain. Biofilms were observed on the epithelial surface (boxes). Scale bar: 50 μm. **Bottom row (E-H).** Representative images of skin samples labeled for the presence of the bacterial DNA binding protein, integration host factor (IHF). Positive labeling for the presence of IHF in bacterial biofilms formed on the epithelial surface is indicated by red fluorescence, epithelium fluoresced green and nuclei appear blue. Scale bar: 10 μm.


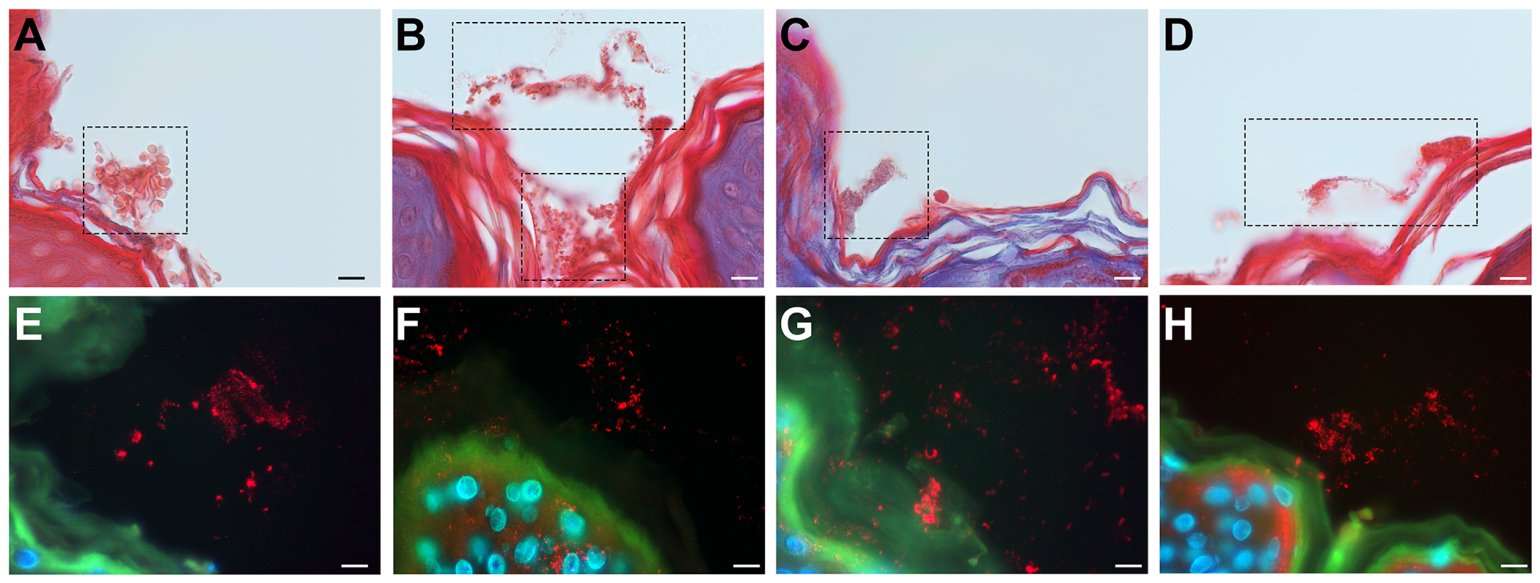


**Supplementary References**

1. . McInnes, P. Manual of Procedures for Human Microbiome Project: Core Microbiome Sampling Protocol A, July 29, 2010. Retrieved on October 10, 2016 from http://www.hmpdacc.org/doc/HMP_MOP_Version12_0_072910.pdf [↑](#endnote-ref-1)
2. . Nadkarni, M.A., Martin, F.E., Jacques, N.A. & Hunter, N. Determination of bacterial load by real-time PCR using a broad-range (universal) probe and primers set. *Microbiology*. **148**, 257-266 (2002). [↑](#endnote-ref-2)
3. . Becerra, S.C., Roy, D.C., Sanchez, C.J., Christy, R.J. & Burmeister, D.M. An optimized staining technique for the detection of Gram positive and Gram negative bacteria within tissue. *BMC Res. Notes*. **9**, 216; 10.1186/s13104-016-1902-0 (2016). [↑](#endnote-ref-3)
4. . Quast, C. *et al*. The SILVA ribosomal RNA gene database project: improved data processing and web-based tools. *Nucleic Acids Res*. **41**, D590-D596 (2013). [↑](#endnote-ref-4)
5. . Rognes, T., Flouri, T., Nichols, B., Quince, C & Mahé, F. VSEARCH: a versatile open source tool for metagenomics. *PeerJ*. **4**, e2584 (2016). [↑](#endnote-ref-5)
6. . Yilmaz, P. *et al*. The SILVA and "All-species Living Tree Project (LTP)" taxonomic frameworks. *Nucleic Acids Res*. **42**, D643-D648 (2014). [↑](#endnote-ref-6)
7. . R Core Team. R: A language and environment for statistical computing. R Foundation for Statistical Computing, Vienna, Austria. ISBN 3-900051-07-0, Available at http://www.R-project.org/ (2012). [↑](#endnote-ref-7)
8. . Grice, E.A. & Segre, J.A. The skin microbiome. *Nat. Rev. Microbiol*. **9**, 244-253 (2011). [↑](#endnote-ref-8)
9. . Clarke, K.R. Non-parametric multivariate analyses of changes in community structure. *Aust. J. Ecol*. **18**, 117-143 (1993). [↑](#endnote-ref-9)
10. . Prescott, S.L. *et al*. The skin microbiome: impact of modern environments on skin ecology, barrier integrity, and systemic immune programming. *World Allergy Organ. J*. **10**, 29; 10.1186/s40413-017-0160-5 (2017). [↑](#endnote-ref-10)
11. . Sanford, J.A. & Gallo, R.L. Functions of the skin microbiota in health and disease. *Semin. Immunol*. **25**, 370-377 (2013). [↑](#endnote-ref-11)
12. . Bouffard, G.G., Blakesley, R.W., Wolfsberg, T.G., Turner, M.L. & Segre, J.A. A diversity profile of the human skin microbiota. *Genome Res*. **18**, 1043-1050 (2008). [↑](#endnote-ref-12)
13. . Roth, R.R. & James, W.D. Microbial ecology of the skin. *Annu. Rev. Microbiol*. **42**, 441-464 (1988). [↑](#endnote-ref-13)
14. . Leeming, J.P., Holland, K.T. & Cunliffe, W.J. The microbial ecology of pilosebaceous units isolated from human skin. *J. Gen. Microbiol*. **130**, 803-807 (1984). [↑](#endnote-ref-14)
